# NEW OPPORTUNITIES FOR ADVANCING DYADIC HEALTH SCIENCE IN GERONTOLOGY

**DOI:** 10.1093/geroni/igad104.1591

**Published:** 2023-12-21

**Authors:** Stephanie Wilson, Joshua Novak, Jeremy Yorgason, Lynn Martire

**Affiliations:** Southern Methodist University, Dallas, Texas, United States; Auburn University, Auburn, Alabama, United States; Brigham Young University, Provo, Utah, United States; Penn State University, State College, Pennsylvania, United States

## Abstract

As dyadic health science enters a golden age, important conceptual, theoretical, and technical challenges remain. Based on a forum review published in The Gerontologist by a group of dyadic scholars, this presentation will provide an overarching framework for understanding the essential components of robust dyadic health research, and the wide array of approaches used within the field. Each component of the framework will be discussed in relation to supporting empirical examples. The presentation will close by identifying crucial challenges and considerations that coincide with important future directions for the field.

